# Environmental Impacts and Their Association With Performance and Excretion Traits in Growing Pigs

**DOI:** 10.3389/fvets.2021.677857

**Published:** 2021-06-21

**Authors:** Alessandra N. T. R. Monteiro, Ludovic Brossard, Hélène Gilbert, Jean-Yves Dourmad

**Affiliations:** ^1^PEGASE, INRAE, Institut Agro, Saint-Gilles, France; ^2^GenPhySE, Université de Toulouse, INRAE, INPT, ENSAT, Castanet-Tolosan, France

**Keywords:** feed efficiency, environmental impacts, life cycle assessment, growing pig, modeling

## Abstract

The selection of pigs for improved production traits has been, for a long time, the major driver of pig breeding. More recently, because of the increasing concern with the environment, new selection criteria have been explored, such as nitrogen (N) excretion. However, many studies indicate that life cycle assessment (LCA) provides much better indicators of environmental impacts than excretion. Therefore, the objective of this study was to investigate, using a modeling approach, the relationships between production traits and LCA impacts of individual growing pigs calculated at the farm gate for 1 kg of body weight gain. Performances of pigs were simulated for 2-phase (2P) and precision feeding (PR), using the InraPorc population model (on 1,000 pigs). Nitrogen excretion was positively correlated with feed conversion ratio (FCR; *r* = +0.96), climate change (CC; *r* = +0.96), acidification potential (AC; *r* = +0.97), eutrophication potential (EU; *r* = +0.97), and land occupation (LO; *r* = +0.96), whatever the feeding program. However, FCR appeared to be a better indicator of LCA impacts, with very high and positive correlations (*r* > +0.99) with CC, AC, EU, and LO for both feeding programs. The CC, AC, and EU impacts of pig production for PR feeding were 1.3, 10, and 7.5% lower than for 2P, respectively, but the correlations within each outcome were very similar among feeding programs. It was concluded that the use of FCR as a selection criterion in pig breeding seems to be a promising approach to associate improved performance and low environmental impact of pig fattening.

## Introduction

The selection of animals for improved production traits has been, for a long time, the major driver of pig breeding (1, 2). More recently, because of the increasing concern with environment, new selection criteria have been explored, such as nitrogen (N) or phosphorus (P) excretion, which are related to both feed efficiency and environmental impact (3). Residual feed intake (RFI) was also proposed as a possible selection criterion to simultaneously improve feed efficiency and reduce N and P excretion (1).

However, the pig supply chain involves a complex system, which requires production of fertilizers and pesticides; production of feed ingredients; feed processing; animal raising; transportation of animals and feed; water use for drinking and cleaning; energy use for light, heat, and ventilation; and waste management (4). Therefore, the environmental degradation is not the consequence of only one process (e.g., the raising of pigs) or one element (e.g., N excretion) and, as reviewed by McAuliffe et al. (4), impacts are better evaluated through integrated methodologies such as life cycle assessment (LCA).

Recently, a comparative LCA showed that pigs selected for low RFI have, on average, 6% lower environmental impacts on climate change (CC), acidification (AC), eutrophication (EU), land occupation (LO), and water depletion than these selected for high RFI (5). However, in this study, RFI did not appear to be the optimum measure for efficient environmentally friendly selection, since it was rather poorly correlated to environmental impacts (*r* = 0.73 for CC in the low RFI line).

The objective of the present study was thus to investigate, using a modeling approach, the relationships between different performance selection traits and LCA environmental impacts evaluated in individual growing pigs.

## Materials and Methods

### Feeding Strategies and Animal Performance

This study considered a conventional growing–finishing pig unit located in West France, as described in detail by Monteiro et al. (6). Two feeds were formulated on the basis of net energy (NE, 9.6 MJ/kg), standardized ileal digestible (SID) amino acids, and digestible phosphorus (P): feed A to achieve 110% the mean population nutrient requirements at the beginning of the growing period (9.84 g/g SID lysine, 3.01 g/kg digestible P), and feed B to achieve 90% the mean population nutrient requirements at the end of the finishing period (4.55 g/kg SID lysine, 1.68 g/kg digestible P). The two feeds were blended according to two feeding programs: 2-phase feeding (2P) corresponding to the strategy used in French central test stations or precision feeding (PR). The 2P pigs were fed with feed A from 30 to 70 kg BW, and then with a blend of 50% of each feed until the end of fattening, to achieve 110% the mean population SID-lysine requirement at the start of the finishing period. For PR pigs, the blend of the two feeds was calculated according to a factorial approach in order that each pig received each day the exact amount of SID lysine required to achieve its potential of protein deposition, which was defined according to a Gompertz function, as described by van Milgen et al. (7).

Simulations for a virtual population of 1,000 female pigs were performed individually, from 30 to 115 kg of BW, for each feeding program to determine individual animal performance, nutrient balance, and excretion according to InraPorc population model (8). This virtual population was generated according to the method described by Brossard et al. (8), from a variance–covariance matrix with two parameters describing individual pig feed intake (the net energy intake at 50 and 100 kg BW: 20.2 ± 2.0 and 25.0 ± 2.9 MJ NE/day, respectively) and three parameters describing the Gompertz function of potential protein deposition (the BW at 70 days: 30.0 ± 2.9 kg, the mean protein deposition rate between 70 days of age and 110 kg BW: 142.8 ± 15.2 g, and the precocity *b-*value of the Gompertz function: 0.0169 ± 0.0103).

The simulated performance and excretion data were then used to calculate gaseous emissions from animals and manure, according to Rigolot et al. (9). The pig production system considered was a conventional growing–finishing pig farm located in Brittany (West France) with indoor raising of animals on complete slatted floor, in a building with mechanical ventilation and collection and storage of manure as liquid slurry (6).

### Life Cycle Assessment

The LCA was performed for each pig, considering all the impacts associated with feed production, animal housing, and manure management (as described by 6). We based our analysis on the CML 2001 (baseline) method version 3.02 as implemented in SimaPro software version 8.05 (PRé Consultants) and added the category land occupation from CML 2001 (all categories) version 2.04. Thus, we considered the potential impacts of pig production on CC (kg CO_2_-eq), EU (g PO_4_-eq), AC (g SO_2_-eq), and LO (m^2^ · year). The CC was calculated according to the 100-year global warming potential factors in kilograms CO_2_-eq. Impacts were calculated at the farm gate, and the functional unit considered was 1 kg of BW gain over the fattening period.

### Statistical Analysis

The LCA calculation model was implemented using SAS software (SAS Inst. Inc., Cary, NC). Performance and environmental impacts were subjected to variance analysis using GLM procedure with feeding strategy as main effect. Pearson correlations for each feeding strategy were calculated between performance and environmental impacts data using CORR procedure, and pigs were ranked according to their CC impact, considering the feeding strategy and using the RANK procedure. All analyses were conducted using SAS software version 9.1 (SAS Inst. Inc., Cary, NC).

All the data used in the statistical analysis are available in the INRAE data repository (10).

## Results and Discussion

### Feeding Strategies, Animal Performance, and Environmental Impacts

Feeding strategies affected most of the parameters evaluated (Table 1); effects were more accentuated for N excretion and N retention efficiency, and for CC, EU, and AC environmental impacts, which are highly dependent on dietary crude protein (CP) content, which was on average lower for PR (144 g/kg) than for 2P (167 g/kg). Compared to 2P, with PR, ADG was slightly improved (by 1.3%), efficiency of N retention was increased (40.5 vs. 36.2%), N excretion was reduced (by 16%), and environmental impacts were decreased (CC, AC, EU, and LO impacts 1.3, 10.0, 7.5, and 0.8% lower than for 2P, respectively). These results are in agreement with previous studies indicating that PR feeding strategy allows the improvement of the performance of pigs, compared to phase feeding, by providing sufficient amount of amino acids even to the animals with the highest potential of protein retention, which may not be the case with phase feeding, especially at the beginning of each phase. In PR compared to 2P feeding strategy, protein and SID lysine intakes were reduced by 9.3 and 22.2%, respectively. Combined with the slightly improved protein retention in PR pigs, this resulted in a significant increase of N retention efficiency (from 36.2 to 40.5%) and a reduction of nutrient load in excreta, contributing to the lower CC, EU, and AC impacts with precision feeding, as already shown by Monteiro et al. (6) and Andretta et al. (11).

**Table 1:** Effect of feeding strategy on pig performance, nitrogen excretion, and environmental impacts measured by life cycle assessment (*n* = 1,000 pigs).

**Item**	**Two-Phase feeding**	**Precision feeding**	***P-*value**^**a**^
**Performance**			
ADFI, g/day	2,310 ± 259	2,316 ± 261	ns
ADG, g/day	864 ± 112	876 ± 116	^*^
FCR, kg/kg	2.69 ± 0.29	2.67 ± 0.32	*t*
SID lysine intake, g/day	1.89 ± 0.20	1.47 ± 0.11	^***^
Protein intake, g/day	375 ± 42.2	340 ± 32.5	^***^
Protein retained, g/day	135 ± 18.4	138 ± 20.1	^*^
N retention efficiency, %	36.2 ± 4.8	40.5 ± 4.8	^***^
**Environmental impacts**			
N excreted, kg/pig	3.83 ± 0.69	3.20 ± 0.56	^***^
CC, kg CO_2_-eq/kg BW gain	2.34 ± 0.25	2.31 ± 0.28	^*^
EU, g PO_4_-eq/kg BW gain	17.4 ± 2.34	16.1 ± 2.22	^***^
AC, g SO_2_-eq/kg BW gain	48.1 ± 7.29	43.3 ± 6.60	^***^
LO, m^2^ year/kg BW gain	3.77 ± 0.40	3.74 ± 0.45	*t*

*ADG, average daily gain; ADFI, average daily feed intake; FCR, feed conversion ratio; CC, Climate Change; EU, Eutrophication potential; AC, Acidification Potential; LO, Land Occupation*.

*Two-phases feeding = pigs received a “growing” diet from 30 to 70 kg of BW and a “finishing” diet from 70 to 115 kg of BW; Precision feeding = individual pigs were fed daily with a diet providing the exact amount of digestible amino acids they required*.

a *t: P < 0.10*;

* *P < 0.05*;

*** *P < 0.001*.

### Correlation Between Performance, Excretion, and Environmental Impacts

**Figure 1:**
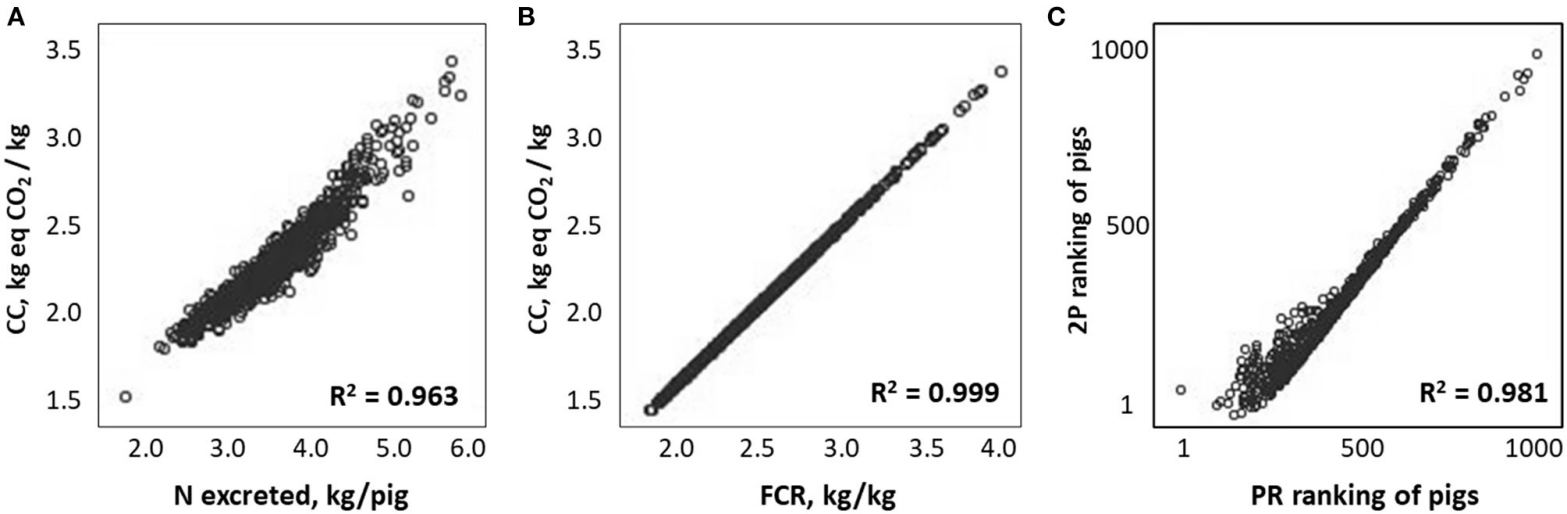
Relationships between climate change (CC) impact and nitrogen excreted **(A)** or feed conversion ratio (FCR, **B**), and effect of feeding program on ranking of pigs according to CC impact **(C)** (*N* = 1,000 pigs).

Correlations between performance, excretion and environmental impacts are shown in Table 2. The correlation values obtained for 2P and PR strategies were very close. Nitrogen excretion was highly and positively correlated with CC (*r* = +0.96, Figure 1A), AC (*r* = +0.97), EU (*r* = +0.97), and LO (*r* = +0.96). Correlations between environmental impacts and NR were much lower than with NE, with *r* values ranging between 0.42 and 0.64, depending on the category. Correlations between environmental impacts and N retention efficiency were similar to these obtained with N excretion.

**Table 2:** Correlations^a^ between performance traits, nitrogen excretion, and environmental impacts, for the precision (PR) and the two-phase (2P, in italic) feeding strategies.

		**ADFI**	**ADG**	**FCR**	**NR**	**NEff**	**NE**	**CC**	**EU**	**AC**
ADG	PR	0.683								
	*2P*	*0.576*								
FCR	PR	0.181	−0.583							
	*2P*	*0.246*	*−0.636*							
NR	PR	−0.692	−0.002	−0.643						
	*2P*	*−0.675*	*−0.112*	*−0.593*						
Neff	PR	−0.361	0.543	−0.986	0.683					
	*2P*	*−0.385*	*0.400*	*−0.959*	*0.669*					
NE	PR	0.187	−0.552	0.963	−0.428	−0.940				
	*2P*	*0.125*	*−0.682*	*0.956*	*−0.450*	*−0.953*				
CC	PR	0.188	−0.577	0.999	−0.647	−0.987	0.963			
	*2P*	*0.249*	*−0.633*	*0.999*	*−0.599*	*−0.960*	*0.955*			
EU	PR	0.225	−0.546	0.998	−0.625	−0.983	0.971	0.998		
	*2P*	*0.225*	*−0.645*	*0.997*	*−0.610*	*−0.973*	*0.966*	*0.997*		
AC	PR	0.241	−0.532	0.996	−0.621	−0.981	0.972	0.996	0.999	
	*2P*	*0.224*	*−0.643*	*0.996*	*−0.617*	*−0.977*	*0.968*	*0.996*	*0.997*	
LO	PR	0.181	−0.583	0.999	−0.643	−0.986	0.963	0.999	0.998	0.996
	*2P*	*0.245*	*−0.637*	*0.999*	*−0.593*	*−0.959*	*0.956*	*0.999*	*0.997*	*0.996*

*ADFI, average daily feed intake; ADG, average daily gain; FCR, feed conversion ratio; NE, nitrogen excretion; CC, Climate Change; EU, Eutrophication potential; AC, Acidification Potential; LO, Land Occupation*.

a *All correlations were significantly different from 0 (P < 0.001)*.

Average daily feed intake (ADFI) presented much lower correlation with all the impact categories (*r* = +0.21 on average). The weak correlation between ADFI and environmental impacts corroborated the 0.25–0.30 values obtained by Soleimani and Gilbert (5).

Feed conversion ratio appeared the best indicator of LCA impacts, with very high and positive correlations (Table 2, *r* > +0.99) with CC (Figure 1B), AC, EU, and LO for both feeding programs. This is consistent with the major contribution of feed intake to most environmental impacts (more than 70% for CC, EU, and LO, and about 30% for AC; 6), as well as to FCR. Moreover, efficient pigs, with lower FCR, ingest less energy and protein per kilogram of gain, which results in reduced enteric and manure methane production, and reduced organic matter, N, and P excretion. Gaseous emissions of N compounds from excreta have an important contribution to CC (due to N_2_O emission) and to AC and EU (due to NH_3_ emission). Moreover, ${\text{NO}}_{3}^{-}$ and ${\text{PO}}_{4}^{-}$ leaching after manure spreading also contributes to EU. This contributes to explain the close correlation between N retention efficiency and environmental impacts (*r* ranging from 0.96 to 0.98 depending on the impact category). These reductions in enteric emissions and emissions from excreta and manure from more efficient pigs (with low FCR) also contribute to explain the close relationship obtained between FCR and environmental impacts, both expressed per kilogram of body weight gain.

Despite the lower CC, AC, EU, and LO of pig production in the PR program, the correlations within each outcome were very similar among feeding programs.

### Between-Animal Variability

It has already been shown that precision feeding strategy removes a constraint on reaching maximum growth potential and allows all animals to express their maximum growth potential, whereas with phase-feeding strategy, the performance of the highest potential animals may be limited due to insufficient amino acid supplies (8, 12). This explains that the variability of performance and environmental impacts may differ according to the feeding strategy. For instance, the coefficient of variation of CC impact was higher with PR than with 2P feeding strategy (12.1 and 10.7%, respectively). This affects the pigs' ranking, as illustrated in Figure 1C, which shows the correlation between the ranking of pigs according to CC impact with the two feeding strategies. Similar results were obtained for FCR.

## Conclusions

The results of this study indicate that FCR is better correlated with environmental impacts evaluated using LCA than nitrogen excretion or other performance criteria. This offers interesting perspectives for the improvement of both feed efficiency and environmental impacts. However, further studies are still required before implementing LCA environmental impacts (or FCR as a proxy of these impacts) in selection programs. The same approach as the one used in this study with simulated data could be carried out on real data collected from selection programs. This would allow the assessment of the genetic parameters of the different LCA impacts and would allow taking better account of all the biological phenomena influencing growth performance, nutrient excretion, and enteric emission, which are probably not completely represented in the growth simulation model. Moreover, the correlated effects on other important criteria, such as carcass lean percentage, meat quality, or animal health and behavior, should also be evaluated.

## Data Availability Statement

All the data used in the statistical analysis are available in the INRAE data repository (10).

## Author Contributions

AM and J-YD contributed to conception and design of the study. AM, LB, and J-YD organized the database. AM, LB, HG, and J-YD wrote sections of the manuscript. All authors contributed to manuscript revision, read, and approved the submitted version.

## Conflict of Interest

The authors declare that the research was conducted in the absence of any commercial or financial relationships that could be construed as a potential conflict of interest.
